# Electronic cigarette exposure disrupts airway epithelial barrier function and exacerbates viral infection

**DOI:** 10.1152/ajplung.00135.2023

**Published:** 2023-09-12

**Authors:** Andjela Raduka, Nannan Gao, Robert L. Chatburn, Fariba Rezaee

**Affiliations:** ^1^Department of Inflammation and Immunity, Lerner Research Institute, Cleveland Clinic Foundation, Cleveland, Ohio, United States; ^2^Enterprise Respiratory Care Research Cleveland Clinic, Cleveland Clinic Children’s, Cleveland, Ohio, United States; ^3^Center for Pediatric Pulmonary Medicine, Cleveland Clinic Children’s, Cleveland, Ohio, United States

**Keywords:** airway epithelial cells, electronic cigarette, epithelial barrier, respiratory syncytial virus (RSV), vaping

## Abstract

The use of electronic cigarettes (e-cigs), especially among teenagers, has reached alarming and epidemic levels, posing a significant threat to public health. However, the short- and long-term effects of vaping on the airway epithelial barrier are unclear. Airway epithelial cells are the forefront protectors from viruses and pathogens. They contain apical junctional complexes (AJCs), which include tight junctions (TJs) and adherens junctions (AJs) formed between adjacent cells. Previously, we reported respiratory syncytial virus (RSV) infection, the leading cause of acute lower respiratory infection-related hospitalization in children and high-risk adults, induces a “leaky airway” by disrupting the epithelial AJC structure and function. We hypothesized chemical components of e-cigs disrupt airway epithelial barrier and exacerbate RSV-induced airway barrier dysfunction. Using confluent human bronchial epithelial (16HBE) cells and well-differentiated normal human bronchial epithelial (NHBE) cells, we found that exposure to extract and aerosol e-cig nicotine caused a significant decrease in transepithelial electrical resistance (TEER) and the structure of the AJC even at noncytotoxic concentrations. Western blot analysis of 16HBE cells exposed to e-cig nicotine extract did not reveal significant changes in AJC proteins. Exposure to aerosolized e-cig cinnamon or menthol flavors also induced barrier disruption and aggravated nicotine-induced airway barrier dysfunction. Moreover, preexposure to nicotine aerosol increased RSV infection and the severity of RSV-induced airway barrier disruption. Our findings demonstrate that e-cig exposure disrupts the airway epithelial barrier and exacerbates RSV-induced damage. Knowledge gained from this study will provide awareness of adverse e-cig respiratory effects and positively impact the mitigation of e-cig epidemic.

**NEW & NOTEWORTHY** Electronic cigarette (e-cig) use, especially in teens, is alarming and at epidemic proportions, threatening public health. Our study shows that e-cig nicotine exposure disrupts airway epithelial tight junctions and increases RSV-induced barrier dysfunction. Furthermore, exposure to aerosolized flavors exaggerates e-cig nicotine-induced airway barrier dysfunction. Our study confirms that individual and combined components of e-cigs deleteriously impact the airway barrier and that e-cig exposure increases susceptibility to viral infection.

## Graphical abstract

**Figure:**
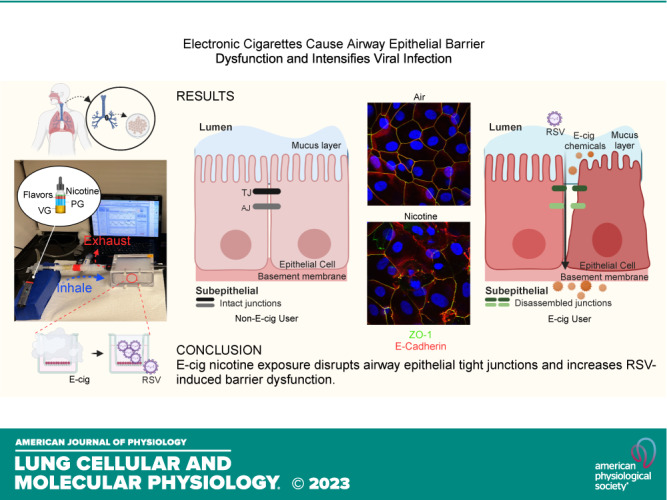

## INTRODUCTION

Electronic cigarettes (e-cigs) are battery-operated devices that aerosolize liquid mixtures of propylene glycol (PG), vegetable glycerin (VG), flavorings, and nicotine. Historically, in 2007, e-cig devices entered the US market and were claimed as a strategy to assist with smoking cessation (1). Since then, there has been a massive expansion of numerous types of e-cig devices and e-liquid formulations. In addition, various flavors, such as fruits, crèmes, cinnamon, and menthol, have been added to the e-liquid, which appeals to the youth market (2). It was in 2019 that numerous cases of e-cig, or vaping, product use-associated lung injury (EVALI) were admitted to the hospitals. In 2020, the Centers for Disease Control and Prevention (CDC) reported 2,807 cases of patients hospitalized with EVALI, which claimed the lives of 68 patients in the United States (3). We published a case series of children and adolescents admitted to Cleveland Clinic Children’s intensive care unit (ICU) with complications of vaping (4). According to the National Youth Tobacco Survey (NYTS), more than 2 million youth in the United States reported current e-cig use in 2021, and over 85% of these individuals reported using flavored e-cigs (5). However, there is potential underreporting of e-cig use due to the implementation of the COVID-19 protocols requiring online sampling. In 2020, when samples were collected on school campuses, more than 3.5 million youth reported the current use of e-cig products (6). Furthermore, the latest NYTS data release from 2022 revealed e-cigs as the most commonly used tobacco product among teenagers since 2014 (7). Despite the recognition of youth e-cigs as an epidemic in the United States and the strong commitment made by the CDC and Food and Drug Administration to support efforts to protect youth from this preventable health risk, the toxicity of e-cigs is not well known (8).

Airway epithelial cells are at the internal and external environment interface and act as the first barrier against inhaled particles, toxins, and viruses (9, 10). Previous studies have shown that exposure to conventional cigarette smoke (CS) disrupts airway epithelial integrity, decreasing transepithelial electrical resistance (TEER) and increasing permeability. One study investigated the effect of e-cig vapor containing nicotine on airway permeability (11). Two other studies measured TEER and permeability post-PG:VG or Crème Br«lée flavor exposure but did not investigate the role of nicotine (12, 13). Of note, neither of these studies investigated the effects of e-cigs on apical junctional complexes (AJCs), a crucial factor regulating the formation and maintenance of the epithelial barrier. AJCs are specialized intercellular structures that form between adjacent cells and consist of tight junctions (TJs) and adherens junctions (AJs), maintaining cell polarity and attachment between neighboring cells (14–16). The apical TJ and AJ comprise multiple proteins such as the claudin family, occludin, tricellulin, junctional adhesion molecules, cadherin family, α-and β-catenin, and zonula occludens (ZO) family (9, 10). AJC dysfunction may perpetuate airway inflammation by facilitating the “outside/in” translocation of inhaled particles, allergens, and pathogens (9, 15). Our published case series of adolescents admitted to our hospital with complications from vaping sparked our passion for studying the impact of e-cig usage on airway epithelial barrier structure and function (4).

Furthermore, Gaiha et al. (17) have shown teenagers and young adults who vaped were more at risk of viral illnesses such as SARS-CoV-2 infection. However, a recent study by Moyers et al. (18) showed no correlation between users and nonusers. A recent clinical study examined the response to a live attenuated influenza virus in nonsmokers and e-cig users. Although there were no significant changes in viral loads between the two groups, e-cig users had suppressed immune responses to the virus, suggesting that e-cig users are at higher risk of viral infection complications than nonsmokers (19). Emerging evidence for the association between e-cig usage and respiratory viral infection also comes from preclinical studies. Exposures to nicotine-free and nicotine-containing e-cig liquid have been shown to promote human rhinovirus infection in primary tracheobronchial epithelial cells derived from young, healthy smokers (20). Moreover, that study used e-cig liquid instead of e-cig aerosol and did not address the impact of e-cigs on the airway epithelial barrier.

Respiratory syncytial virus (RSV) is the leading cause of acute lower respiratory tract infection (ALRI) in young children and a significant cause of respiratory illness in high-risk adults and the elderly population (9, 21–24). Previous work in our laboratory showed that RSV diminishes the integrity of the epithelial barrier by disrupting AJC (25). We also showed coexposure to titanium dioxide nanoparticles (TiO_2_-NP), which we used as an air pollution model, increased airway inflammation and exaggerated RSV-induced epithelial barrier dysfunction in both airway epithelial cell cultures and a mouse model (26). The combined exposure to e-cigs and other environmental agents may predispose e-cig users to more clinically significant lung damage. However, little is known about the impact of e-cig exposure on airway epithelial cell integrity. We hypothesized that e-cig exposure disrupts AJC structure and function and exacerbates RSV-induced airway injury and inflammation. To test this hypothesis, we examined the effects of the PG, VG, nicotine, and flavoring components of e-cig liquid on airway epithelial barrier function and structure using our well-established in vitro model.

## MATERIALS AND METHODS

### Antibodies

The following primary monoclonal (mAbs) and polyclonal (pAbs) antibodies were used to detect epithelial apical junctional complex proteins by immunofluorescent labeling and/or immunoblotting. Tight junction: ZO-1 (Invitrogen, Cat. No. 33–9100, Waltham, MA) and occludin (Invitrogen, Cat. No. 33–1500, Waltham, MA); Adherens junction: β-catenin and E-cadherin (Abcam, Cat. No. ab32572, Cat. No. ab40772, Cambridge, MA, and BD Biosciences, Cat. No. 610153, Cat. No. 610181, San Jose, CA). Cleaved caspase-3 (Asp175) (Cat. No. 9661) was purchased from Cell Signaling Technology (Danvers, MA). Anti-rabbit and anti-mouse secondary antibodies conjugated to Alexa Fluor 488 and 568 (Thermo-Fisher Scientific Cat. No. A21206, RRID:AB_2535792; Cat. No. A10042, RRID:AB_2534017; Cat. No. A21070, RRID:AB_2535731; Cat. No. A21202, RRID:AB_141607) were also obtained for immunofluorescence staining. Anti-rabbit (Thermo-Fisher Scientific Cat. No. 31460, RRID:AB_228341) and anti-mouse (Thermo-Fisher Scientific Cat. No. 31430, RRID:AB_228307) secondary antibodies conjugated to horseradish peroxidase were purchased for Western blotting. Glyceraldehyde 3-phosphate dehydrogenase (GAPDH) (Cat. No. ab8245) was obtained from Abcam (Cambridge, MA). Detailed information, including clone number, validation, application, and dilution, was included in Table 1.

**Table 1.** Primary antibodies used for immunostaining and Western blot

Antibodies	Source	Cat. No.	Clone Number	Validation	Working Dilution
ZO-1	Invitrogen	33-9100	1A12	doi: 10.1074/jbc.M114.556449	1:300 (IF) 1:1,000 (WB)
E-cadherin	Abcam	ab40772	EP700Y	doi.org/10.1242/dev.172791	1:300 (IF)
E-cadherin	BD Biosciences	610181	36	doi: 10.1371/journal.pgen.1008451	1:300 (IF) 1:2,000 (WB)
Occludin	Invitrogen	33-1500	OC-3F10	doi: 10.1248/bpb.b15-01023	1:300 (IF) 1:2,000 (WB)
β-catenin	Abcam	ab32572	E247	doi: 10.1002/hep.30270	1:300 (IF)
β-catenin	BD Biosciences	610153	14	doi: 10.1038/s42003-020-0916-2	1:2,000 (WB)
GAPDH	Abcam	ab8245	6C5	doi: 10.1038/s41467-018-08187-6	1:50,000 (WB)
Cleaved Caspase-3 (Asp175)	Cell Signaling Technology	9661	Polyclonal Antibody	doi: 10.1128/JVI.01573-13	1:1,000 (WB)

IF, immunofluorescence staining; WB, Western blot.

### Chemicals and Reagents

Propylene glycol (PG) and vegetable glycerin (VG) were purchased from Biopharm. Nicotine (Cat. No. N3876) was purchased from Sigma-Aldrich (St. Louis, MO). The following e-liquids used in the aerosolized exposure experiments were purchased from local vape shops (January 2022–January 2023), all in 50%/50%, PG:VG (50:50):unflavored nicotine (36 mg/mL) (Air Factory E-Liquid, Irvine, CA, Lot. No. 1040861), menthol-flavored nicotine (36 mg/mL) (Air Factory E-Liquid, Irvine, CA, Lot. No. 1035284), menthol-flavored e-liquid (Halo, Gainesville, FL, Lot. No. B500201203B05), cinnamon-flavored e-liquid, cinnamon-flavored nicotine (2.4%), and cinnamon-flavored nicotine (4.8%) (Monster Vape Labs, FL, Lot. No. B5216-001, FF631-001, and 5D5CO-001 respectively).

### Cell Culture

#### Human bronchial epithelial cells.

16HBE14o- human bronchial epithelial (16HBE) simian virus 40 (SV-40)-immortalized bronchial epithelial cell line was generously donated by the late Professor Dieter Gruenert, University of California, San Francisco, also commercially available through American Type Culture Collection. 16HBE is a well-characterized and manipulable human bronchial epithelial cell line, which under submerged culture conditions, forms monolayers that exhibit well-defined TJ and AJ (27–29). Cells were grown on collagen-coated transwell permeable membranes (Corning, Tewksbury, MA) or cultured on flat-bottom well plates under liquid-liquid conditions (26, 30, 31). Type 1 rat tail collagen was purchased from BD Biosciences (San Jose, CA). Cells were cultured in Dulbecco’s minimal essential medium (DMEM, Cat. No. 11995, Gibco, Waltham, MA) supplemented with 0.5% antibiotic-antimycotic (Cat. No. 15240-062, Gibco), 10% heat-inactivated fetal bovine serum (Cat. No. 35-011-CV, Corning), and HEPES (Cat. No. 15630, Gibco). At full confluency, cells were exposed to e-cig extract under liquid-liquid conditions or e-cig aerosol by reducing the apical chamber media to 30 µL for direct exposure. All cell lines were authenticated before experiment initiation.

#### Normal human bronchial epithelial cells.

Cells were isolated from the lungs of normal donors and were grown on transwell permeable membranes with defined media [DMEM/Ham’s F12 medium supplemented with 50 µg/mL gentamicin (Cat. No. 15750060, Thermo-Fisher Scientific), 0.5% antibiotic-antimycotic (Cat. No. 15240-062, Gibco), and 2% Ultroser G serum substitute (Cat. No. 15950-017, Sartorius, Edgewood, NY)]. They were differentiated under air-liquid interface (ALI) conditions by removal of medium from the apical surface and as previously described (26, 27, 30–32). At full confluency, polarized NHBE cells grown in ALI were exposed directly to aerosolized e-cig chemicals.

### Transepithelial Electrical Resistance

16HBE and NHBE cells (50,000 cells/well) were cultured on 24-well transwell inserts (Corning Cat. No. 3470) until the cells were completely confluent. Cells were exposed to either e-cig extract or aerosol. Transepithelial electric resistance (TEER) was evaluated using an EVOM2 voltohmmeter (World Precision Instruments, Sarasota, FL) and shown as the percent change from time zero as per previous studies (31, 33). Experiments were performed using cell monolayers with a TEER > 500 Ω×cm^2^. Data points were then compared with the control.

### E-Cig Chemical Preparation for Extract Exposure

All e-cig liquids were freshly prepared or purchased. PG and VG were mixed with various ratios and vortexed till combined, and the resulting mixture was referred to as PG:VG ratios. PG:VG was tested in different volume ratios and concentrations (% in culture media). For extract exposures, nicotine was directly dissolved into PG:VG.

### E-Cigarette Aerosol Exposure

We used an ASL 5000 breathing simulator (IngMar Medical, Pittsburgh, PA), which generates realistic patients’ breathing patterns. The equipment works with specialized software that uses preprogrammed lung models with various tidal volumes, breath rates, and realistic inspiratory to expiratory time ratios (I:E). The software was set to simulate e-cig puffs and normal breathing patterns of 13–18-yr-old teenagers. A custom-built air-tight cell chamber (Prototype Core, Cleveland Clinic) with a volume of 400 mL was connected to the IngMar ASL 5000 breathing simulator to represent the upper airways (Fig. 4). An anesthesia bag in a rigid cylinder (IngMar Auxiliary Gas Exchange Cylinder) was connected between the cell chamber and the ASL 5000 to keep vaping aerosol from contaminating the breathing simulator. A three-way valve was used to connect a fourth-generation e-cig device (Vaporesso XROS set at 1.2Ω, power 11 W/16 W) and HEPA filter to the air-tight cell chamber. A separate group of cells was exposed to HEPA-filtered air (control) with the same puff pattern. Before exposure, polarized cell culture plates growing at ALI (NHBE) or with reduced media (16HBE) were placed inside the cell chamber. The puff was set to 4 puffs/min lasting for 3 s, with a volume of 100 mL based on previous publications (11, 12, 34–37). Each puff was followed by two normal breaths, and the breath rate was set to 12 per minute for a total exposure time of 15 min. At the end of exposure, cell culture plates were left in the cell chamber for 5 min to allow for equilibration before returning to the incubator (37°C, 5% CO2). Experiments were repeated at indicated time points, and cells were harvested at the end of experiments. TEER was measured at *time 0* and indicated times postexposure, normalized to *time 0*, and compared with the HEPA-filtered air group. The e-cig exposure was approved by Cleveland Clinic Environmental Health and Safety (EHS).

### Respiratory Syncytial Virus

rrRSV derived from RSV A2 strain, a recombinant expressing red fluorescent protein (RFP) upon replication, was a kind gift from Dr. Mark Peeples (Nationwide Children’s Hospital Research Institute, Columbus, OH) and Dr. Peter Collins (National Institute of Health, Bethesda, MD) (26, 30, 31, 38). RSV was cultured and collected as previously described (32, 39). RSV infection [A2 strain, at a multiplicity of infection (MOI) of 0.25] of 16HBE cells was performed 24 h after e-cig exposure.

### Cytotoxicity Assay/Lactate Dehydrogenase Assay

Lactate dehydrogenase (LDH) assay was performed as previously described (26, 29, 32). Briefly, the cell culture medium was collected and centrifuged to remove cell debris. Negative control was defined as LDH in the cell culture medium. Positive control was defined as LDH release with exposure to 2% Triton X-100. The measurement of LDH release in the cell culture supernatant was determined by a commercially available LDH Cytotoxicity Detection kit (Takara Bio, Mountain View, CA). The cytotoxicity index was calculated using the following equation:

$$\text{Cytotoxicity index}=\frac{(\text{sample }-\text{ negative control})\;}{\left(\text{positive control }-\text{ negative control}\right)}$$

### Immunofluorescence Labeling and Confocal Microscopy

Cell monolayers cultured on transwell inserts were fixed in cold methanol. For immunofluorescent labeling of TJ and AJ proteins, cell monolayers were incubated with the antibodies mentioned above. Briefly, membranes were blocked with 5% bovine serum albumin in PBS, followed by primary antibody incubation. Membranes were then rinsed three times in PBS, followed by secondary antibody incubation. Furthermore, membranes were rinsed three times in PBS, stained with DAPI, and mounted with an anti-fading mounting medium (Vector Laboratories, Cat. No, H-1000, Burlingame, CA) (26, 30, 31). Samples were imaged with ×63 objectives using a Leica TCS-SP8-AOBS inverted confocal microscope (Leica Microsystems, Wetzlar, Germany). Images were processed with Adobe Photoshop (RRID:SCR_014199) software.

### Western Blot Analysis

Cell lysates were collected after exposure with RIPA lysis buffer (with Halt protease and phosphatase inhibitors, Thermo Scientific, Waltham, MA). Western blot analysis was performed as previously described (30, 31). Briefly, total protein concentrations were determined by the Pierce BCA Protein Assay kit (Thermo Scientific, Waltham, MA). This was followed by SDS-PAGE and wet or dry transfer to polyvinylidene difluoride (PVDF) membranes (Bio-Rad Laboratories, Hercules, CA). Membranes were incubated with specific primary antibodies left overnight at 4°C, followed by secondary antibodies with horseradish peroxidase for 1 h at room temperature. Blots were visualized with regular or enhanced chemiluminescence (ECL) substrates (Thermo Scientific, Waltham, MA), and immunoreactive bands were imaged using ChemiDoc Imaging Systems (Bio-Rad Laboratories, RRID:SCR_008426). The density of each band was measured with ImageJ software (RRID:SCR_003070) and normalized to the lane-loading control, mouse anti-GAPDH (Abcam, Cambridge, UK).

### Statistical Analysis

Data were analyzed using Prism software (GraphPad Prism, RRID:SCR_002798). Data are presented as the mean and standard error of the mean (SE) and are representative of three or more experiments. For the comparison of multiple groups, statistical analysis was performed using one-way ANOVA when comparing more than two groups, followed by Dunnett’s post hoc test for all groups of the experiment. A *t* test was used when comparing the difference between the two groups. Statistical difference was considered when *P* < 0.05.

## RESULTS

### E-Cig Solvent, PG:VG, Decreased Barrier Integrity and Induced AJC Disassembly

**Figure 1.**
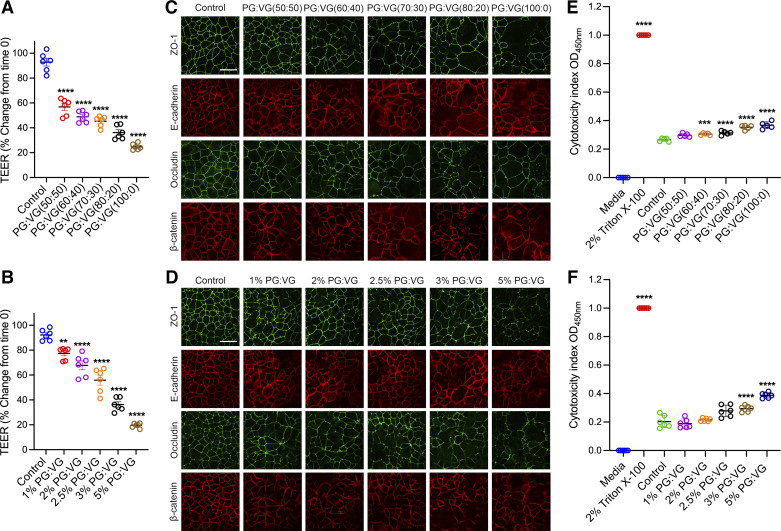
Exposure to PG:VG induced barrier dysfunction and AJC disassembly. Confluent 16HBE cells were exposed to control medium or various PG:VG ratios (2.5% vol/vol at 50:50, 60:40, 70:30, 80:20, and 100:0) and concentrations (1%, 2%, 2.5%, 3%, and 5% vol/vol at 50:50 ratio) for 24 h. *A*: at 24 h postexposure to various PG:VG ratios, TEER (Ω × cm^2^) was measured by the volt-ohm meter and plotted as percentage versus *time 0* for each group. *B*: at 24 h postexposure to various PG:VG (50:50) concentrations, TEER was measured and plotted as a percentage versus *time 0* for each group. *C*: at 24 h postexposure, cells were fixed with methanol and immunolabeled for tight junction proteins (ZO-1 and occludin) and adherens junction proteins (E-cadherin and β-catenin) and imaged by confocal microscopy. *D*: at 24 h postexposure, cells were fixed with methanol and immunolabeled for tight junction proteins (ZO-1 and occludin) and adherens junction proteins (E-cadherin and β-catenin) and imaged by confocal microscopy. The cytotoxicity index of cells exposed to different PG:VG ratios (*E*) and concentrations (*F*) for 24 h. The culture medium served as the negative control, and the medium from cells treated with 2% Triton X-100 served as the positive control. The cytotoxicity index was calculated and compared with untreated cells (control). Data are shown as means ± SE, *n* = 5-6 independent experiments; one-way ANOVA followed by Dunnett’s multiple comparisons test. **P < 0.01, ***P < 0.001, ****P < 0.0001. The images are representative of at least three independent experiments. Scale bar, 30 μm. AJC, apical junctional complex; PG, propylene glycol; TEER, transepithelial electrical resistance; VG, vegetable glycerin.

PG:VG has been used as a solvent for e-liquid components to add flavor and viscosity. It has been shown that PG:VG is toxic when administered at higher concentrations to the cell culture medium (13, 40, 41). Here, we aimed to investigate the effects of PG:VG on airway epithelial cell function by measuring TEER. This physiologically relevant value can be measured in real-time without damaging cell layers. 16HBE cells were cultured on semipermeable membrane inserts and allowed to become confluent, with an average TEER of >500 Ω × cm^2^. We added the PG:VG compounds in the e-cig liquid directly to the cell culture media for 24 h. Our studies used five different e-liquid PG:VG ratios used in e-liquids products. 16HBE cells were exposed to PG:VG ratios: 50% PG and 50% VG (50:50), 60% PG and 40% VG (60:40), 70% PG and 30% VG (70:30), 80% PG and 20% VG (80:20), and 100% PG and 0% VG (100:0) (Fig. 1*A*). We found that cells exposed to higher ratios of PG showed more significant decreases in TEER compared with the lower ratios. Based on this set of studies, we chose the PG:VG (50:50) ratio because it caused a decrease in TEER without significant toxicity. In addition, PG:VG (50:50) ratio is the most commonly sold in vape shops and consumed among e-cig users and has been frequently used by previous studies to evaluate the impact of e-cig extract on cell cultures (13, 42–45). 16HBE cells were exposed to 50%/50% PG:VG (50:50) at a concentration of 1–5% (vol/vol). We observed a concentration-dependent decrease in TEER caused by PG:VG (Fig. 1*B*). To study AJC structure, control and treatment groups were subjected to immunofluorescence labeling of TJ proteins (ZO-1 and occludin) and AJ proteins (E-cadherin and β-catenin). Control, untreated epithelial cells demonstrated well-defined continuous junctional labeling. However, cells treated with different ratios of PG:VG showed a dose-dependent increase in disruption as the ratio of PG increased (Fig. 1*C*). Furthermore, cells treated with different concentrations of 50:50 PG:VG revealed a concentration-dependent disruption of the AJC, evidenced by discontinuous labeling at the cell-cell contact areas (Fig. 1*D*). Next, we evaluated the cytotoxicity of different PG:VG ratios and concentrations by measuring the release of LDH into the extracellular space. Cell culture medium from 16HBE cells exposed to PG:VG was collected. The medium from 2% Triton X-100-treated cells was used as a positive control, and the fresh cell culture medium as a negative control. Compared with medium from untreated cells (control), we observed cytotoxicity in cells treated with higher PG ratios (Fig. 1*E*) and higher concentrations of PG:VG exposure (Fig. 1*F*). Based on these sets of experiments, we chose 2.5% PG:VG (50:50) for the following e-cig experiments with added nicotine and flavoring because this dose induced significant AJC disruption and decreased the integrity of the airway epithelial barrier without apparent toxicity.

### Exposure to E-Cig Nicotine Extract Decreased Barrier Integrity and Induced AJC Disassembly

**Figure 2.**
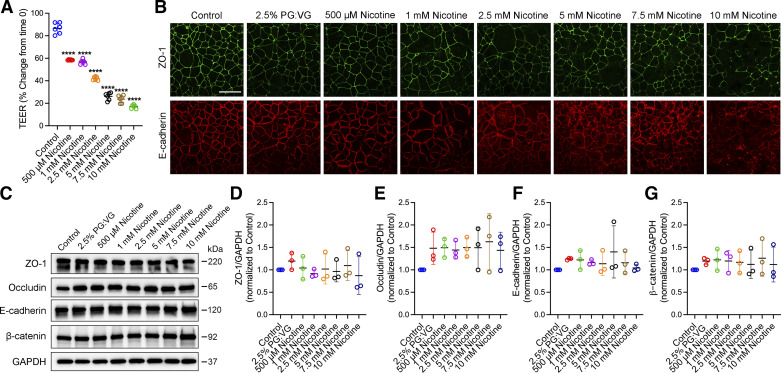
Exposure to e-cig nicotine extract disrupted the airway epithelial barrier in a dose-dependent manner. Confluent 16HBE cells were exposed to a control medium or multiple concentrations of nicotine extract (0.5–10 mM) prepared in 2.5% PG:VG (50:50). *A*: at 24 h postexposure, TEER (Ω × cm^2^) was measured by the volt-ohm meter and plotted as a percentage change from *time 0*. *B*: cells were fixed with methanol and immunolabeled for tight junction protein (ZO-1) and adherens junction protein (E-cadherin) and imaged by confocal microscopy. *C*: representative Western blot of whole cell lysates of three independent experiments from 16HBE cells exposed to control medium, 2.5% PG:VG (50:50), and various nicotine extract concentrations for 24 h. GAPDH served as the control for protein loading. Densitometric qualification of three independent experiments for AJC components ZO-1 (*D*), occludin (*E*), E-cadherin (*F*), and β-catenin (*G*) Western blotting. Data are shown as means ± SE, *n* = 3–6, one-way ANOVA followed by Dunnett’s multiple comparisons test. ****P < 0.0001. Images are representative of three independent experiments. Scale bar, 30 μm. AJC, apical junctional complex; PG, propylene glycol; TEER, transepithelial electrical resistance; VG, vegetable glycerin; 16HBE, 16HBE14o- human bronchial epithelial.

Next, we investigated the effects of e-cig nicotine exposure on AJC structure and function by adding e-cig nicotine extract to the culture media at different concentrations. Polarized 16HBE cell monolayers were grown to confluent and exposed to media control, 2.5% PG:VG (50:50), and 0.5–10 mM nicotine extract prepared in 2.5% PG:VG (50:50). Measuring TEER at 24 h showed that the resistance of cells treated with nicotine significantly decreased in a dose-dependent manner compared with the control cells (Fig. 2*A*). Immunofluorescence labeling and confocal analysis of subcellular localization of TJ (ZO-1) and AJ (E-cadherin) proteins were performed at 24 h postexposure. Control untreated cells showed a normal “chicken wire” appearance of intact TJs and AJs, whereas cells treated with e-cig nicotine exhibited a dose-dependent disruption of the TJs and AJs, with gaps and breaks in junctional strands (Fig. 2*B*). To examine the effect of nicotine on the expression of AJC proteins, we measured the protein levels of several key TJ and AJ proteins. The Western blot densitometric quantification of whole cell lysates showed no significant differences in the protein expression levels of TJ and AJ proteins among control-medium treated cells, 2.5% PG:VG (50:50), and various nicotine extract concentration groups (Fig. 2, *C*–*G*). These observations suggest that nicotine exposure induced disruption of the airway epithelial barrier by mediating TJ and AJ protein disassembly and not by changes in their protein expression.

### Exposure to Low Concentrations of E-Cig Extract Did Not Induce Cell Cytotoxicity or Apoptosis

**Figure 3.**
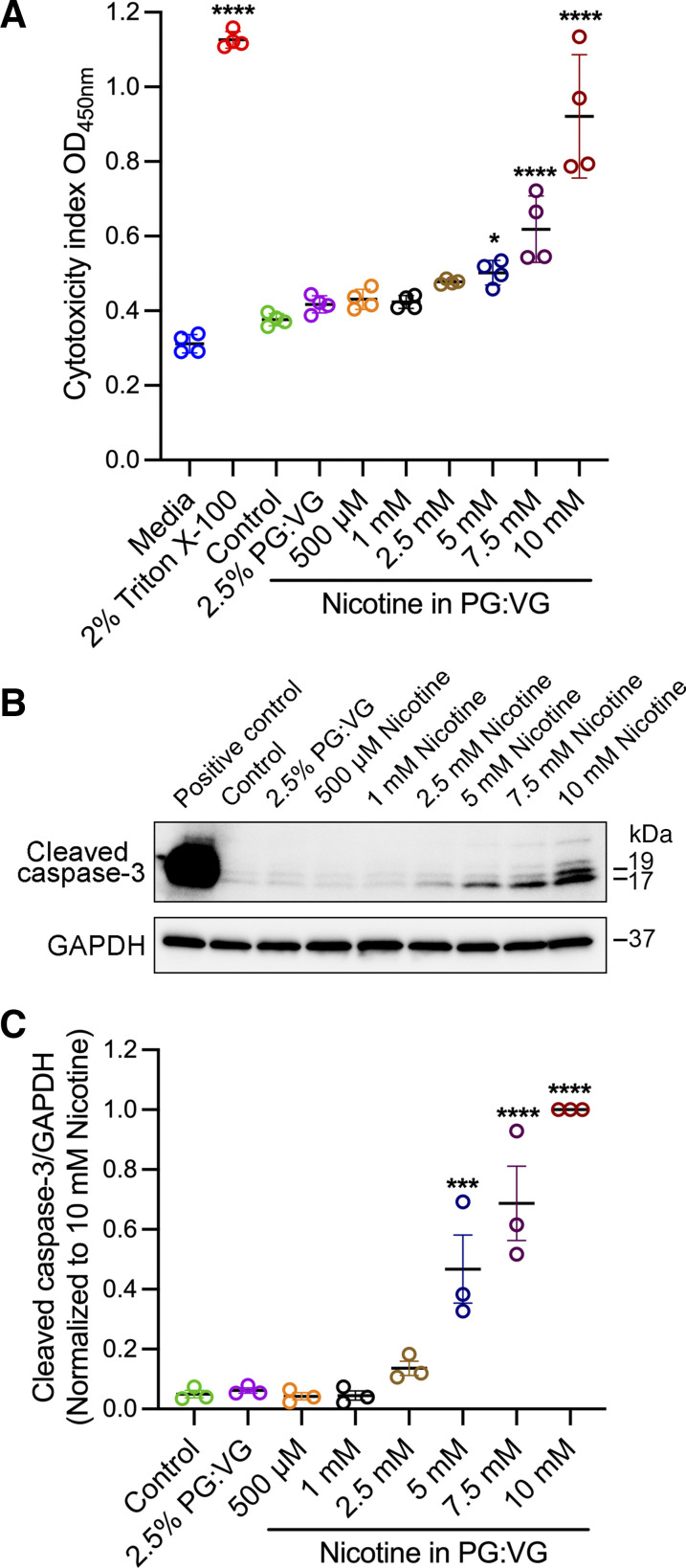
Exposure to lower concentrations of e-cig extract did not cause cell cytotoxicity or apoptosis. Confluent 16HBE cells exposed to control medium, 2.5% PG:VG (50:50), or various concentrations of nicotine (0.5–10 mM) prepared in 2.5% PG:VG (50:50) for 24 h. *A*: results show the cytotoxicity index of cells exposed to various nicotine extract concentrations for 24 h compared with untreated cells. The culture medium served as a negative control, and cells lysed with 2% Triton X-100 served as a positive control. *B*: representative Western blot of whole cell lysates from 16HBE cells of three independent experiments probed with antibodies against cleaved caspase-3. Cell lysate from cytochrome c-treated Jurkat cells served as the positive control. GAPDH served as the control for protein loading. Total protein levels of cleaved caspase-3 (*C*) were analyzed by densitometry and plotted as normalized as indicated. Data are presented as means ± SE, *n* = 3 or 4, one-way ANOVA followed by Dunnett’s multiple comparisons test. *P < 0.05, ***P < 0.001, ****P < 0.0001. Images are representative of three independent experiments. 16HBE, 16HBE14o- human bronchial epithelial.

To determine the cytotoxicity of different concentrations of nicotine extract, we measured the release of LDH from 16HBE cells exposed to different concentrations of e-cig nicotine for 24 h. We used 2% Triton X-100 as the positive control and cell culture medium as the negative control. The cytotoxicity index for each condition was calculated and compared with untreated cells (control). Consistent with previous studies, we observed cytotoxicity at higher concentrations of e-cig nicotine extract exposure (Fig. 3*A*). In an independent study, we analyzed whole cell lysates using Western blotting with antibodies recognizing total and cleaved caspase-3, a cell apoptosis marker. Consistent with the results from the LDH assay, we observed an increase in the expression of cleaved caspase-3 protein in cells treated with 5, 7.5, and 10 mM nicotine extract compared with the control (Fig. 3, *B* and *C*) but not in cells treated with concentrations between 0.5, 1, and 2.5 mM.

### Exposure to Aerosolized E-Cig Nicotine Caused AJC Disassembly and Decreased Barrier Integrity, Similar to the Nicotine Extract

**Figure 4.**
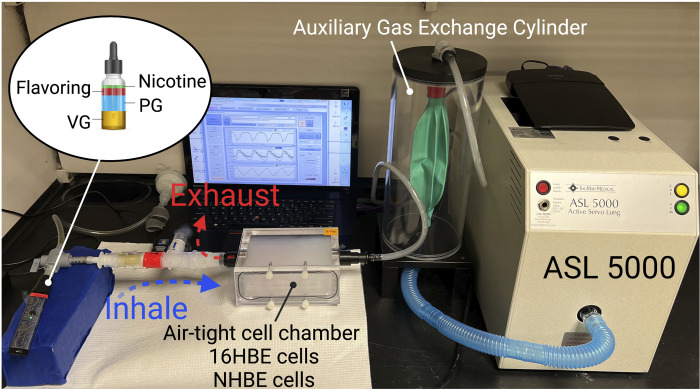
Schematic diagram of experiment model for e-cig aerosol exposure. IngMar ASL 5000 breathing simulator that generates realistic patient breathing patterns is connected to a laptop with specialized software that uses preprogrammed lung models with various tidal volumes and breath rates. A custom-built air-tight cell chamber is connected to the ASL 5000 to represent the upper airways. An anesthesia bag in a rigid cylinder (IngMar Auxiliary Gas Exchange Cylinder) connects the cell chamber and the ASL 5000 to keep vaping aerosol from contaminating the breathing simulator. A three-way valve connects the e-cig (Vaporesso XROS vaping pen) and HEPA filter to the air-tight cell chamber. The annotation of the photograph was created with a licensed version of BioRender.com.

**Figure 5.**
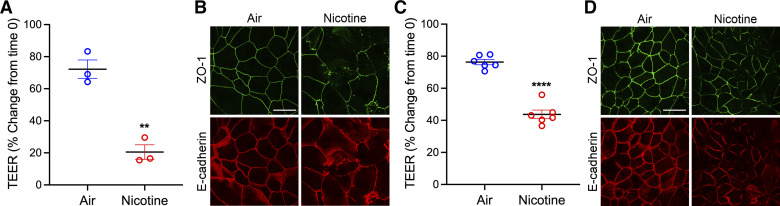
Exposure of differentiated NHBE and polarized 16HBE cells to e-cig nicotine aerosol disrupted the airway barrier. *A*: NHBE cells were grown on permeable membranes, differentiated under an air-liquid interface, and exposed to 36 mg/mL aerosolized e-cig nicotine or HEPA-filtered air at 0, 8, 24, 32, and 48 h. TEER (Ω × cm^2^) was measured by the volt-ohm meter at 48 h postexposure and plotted as a percentage change from *time 0*. *B*: after 48 h of aerosolized e-cig nicotine exposure, NHBE cells were fixed with methanol and immunolabeled for tight junction protein (ZO-1) and adherens junction protein (E-cadherin) and imaged by confocal microscopy. *C*: 16HBE cells were grown to confluent and exposed to 36 mg/mL aerosolized e-cig nicotine or HEPA-filtered air at 0, 8, and 24 h. TEER was measured at 24 h postexposure and plotted as a percentage change from *time 0*. *D*: after 24 h postexposure of 16HBE cells to aerosolized e-cig nicotine, cells were fixed with methanol and immunolabeled for tight junction protein (ZO-1) and adherens junction protein (E-cadherin) and imaged by confocal microscopy. Data are shown as means ± SE, *n* = 3 or 6, unpaired *t* test. **P < 0.01, ****P < 0.0001. The images are representative of at least three independent experiments. Scale bar, 30 μm. NHBE, normal human bronchial epithelial; TEER, transepithelial electrical resistance; 16HBE, 16HBE14o- human bronchial epithelial.

Nicotine salts are compounds used widely in e-cig liquid. Compared with regular nicotine, nicotine salts provide a smoother hit, deliver higher nicotine concentrations, and produce a dense vapor (46, 47). Here, we sought to investigate the impact of aerosolized e-cig nicotine on the structure and function of airway epithelial cells. To do this, we built a customized in vitro delivery system to mimic youth consumers’ puff volume and breathing patterns (the model has been illustrated in the materials and methods and Fig. 4). Briefly, we used an ASL 5000 breathing simulator (IngMar Medical, Pennsylvania), which generates puffs and normal breathing, imitating normal inhalation and exhalation. We designed a program with the ASL software to simulate e-cig puffs and normal breathing patterns of 13–18-yr-old teenagers. NHBE cells isolated from a 13-yr-old male donor were grown and differentiated at an air-liquid interface (ALI) on transwell inserts placed inside a custom-built air-tight cell chamber. NHBE cell monolayers were exposed to 36 mg/mL e-cig nicotine aerosol or HEPA-filtered air, as described in the materials and methods. The treatment was repeated at 8 h, 24 h, 32 h, and 48 h. The TEER was measured at *time 0* and every 3 h postexposure, normalized to *time 0*, and compared with the control HEPA-filtered air group. When using 16HBE, the apical chamber media was reduced to 30 µL to facilitate direct aerosol exposure, and the e-cig aerosol was administrated at 0 h, 8 h, and 24 h. In both NHBE and 16HBE cells, aerosolized e-cig nicotine significantly decreased TEER (Fig. 5, *A* and *C*). At the end of the procedure, cells were fixed with methanol for immunofluorescence labeling of TJ (ZO-1) and AJ (E-cadherin) proteins, followed by the analysis of their subcellular localization using confocal microscopy. In NHBE and 16HBE, the TJ and AJ were predominantly localized at the membrane with a normal “chicken-wire” pattern of intact AJs and TJs. In contrast, this immunolabeling pattern was markedly disrupted in aerosolized e-cig nicotine-exposed cells (Fig. 5, *B* and *D*).

### Aerosolized E-Cig-Flavored Exposure Induced AJC Disassembly and Exaggerated E-Cig Nicotine-Induced Airway Barrier Dysfunction

**Figure 6.**
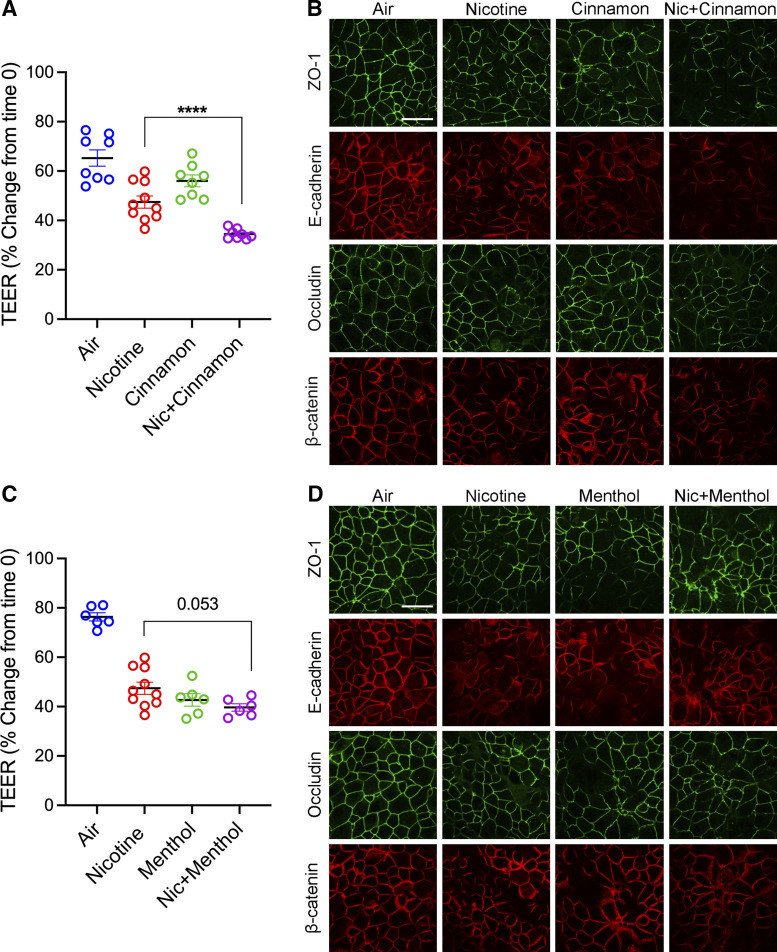
Exposure to flavor in e-cig liquid induced barrier dysfunction and exaggerated e-cig nicotine-induced airway barrier dysfunction. *A*: confluent 16HBE cells were exposed to aerosolized e-cig nicotine, cinnamon, or e-cig nicotine mixed with cinnamon or HEPA-filtered air three times for 24 h. TEER (Ω × cm^2^) was measured 24 h postexposure and plotted as a percentage change from *time 0*. *B*: at 24 h postexposure, 16HBE cells were fixed with methanol and immunolabeled for tight junction proteins (ZO-1 and occludin) and adherens junction proteins (E-cadherin and β-catenin) and imaged by confocal microscopy. *C*: confluent 16HBE cells were exposed to aerosolized e-cig nicotine, menthol, or e-cig nicotine mixed with menthol or HEPA-filtered air three times for 24 h. TEER was measured at 24 h postexposure and plotted as a percentage change from *time 0*. *D*: at 24 h postexposure, 16HBE cells were fixed with methanol and immunolabeled for tight junction proteins (ZO-1 and occludin) and adherens junction proteins (E-cadherin and β-catenin) and imaged by confocal microscopy. Data are presented as means ± SE, *n* = 6–10, one-way ANOVA followed by Dunnett’s multiple comparisons test. ****P < 0.0001. The images are representative of at least three independent experiments. Scale bar, 30 μm. TEER, transepithelial electrical resistance; 16HBE, 16HBE14o- human bronchial epithelial.

Next, we investigated the impact of added aerosolized e-cig flavors on the epithelial barrier structure and function. 16HBE were grown to confluent and exposed to aerosolized e-cig flavors (cinnamon or menthol) with or without 36 mg/mL nicotine or HEPA-filtered air using the air-tight cell chamber and ASL 5000 breathing simulator. Aerosol exposure was performed at 0 h, 8 h, and 24 h. TEER measured epithelial barrier function, and barrier structure was examined by analyzing subcellular localization using immunofluorescence labeling of TJ (ZO-1 and occludin) and AJ (E-cadherin and β-catenin) proteins followed by confocal microscopy. Cells exposed to aerosolized e-cig cinnamon or menthol flavor showed a significant decrease in TEER compared with cells exposed to HEPA-filtered air (Fig. 6, *A* and *C*). Similarly, immunofluorescent labeling showed significant disruption of the TJs and AJs in cells exposed to cinnamon or menthol flavor (Fig. 6, *B* and *D*). Furthermore, epithelial cells exposed to both e-cig nicotine and cinnamon flavor showed a significant decrease in TEER when compared with cells individually exposed to e-cig nicotine or cinnamon (Fig. 6*A*). Consistently, immunofluorescent labeling of epithelial cells exposed to e-cig nicotine and cinnamon flavor exhibited a marked increase in disassembly of TJ and AJ structure (Fig. 6*B*). In contrast, the TEER was slightly lower in cells exposed to both e-cig nicotine and menthol compared with cells that were only exposed to e-cig nicotine or menthol (Fig. 6*C*). Immunofluorescent labeling of TJs and AJs showed an increase in disassembly of chicken-wire pattern in cells exposed to both e-cig nicotine and menthol compared with individual exposure (Fig. 6*D*). Our results showed that adding specific flavors in today’s market has detrimental effects on airway epithelial cell barrier formation.

### E-Cig Nicotine Exacerbated RSV-Induced Airway Barrier Disruption

**Figure 7.**
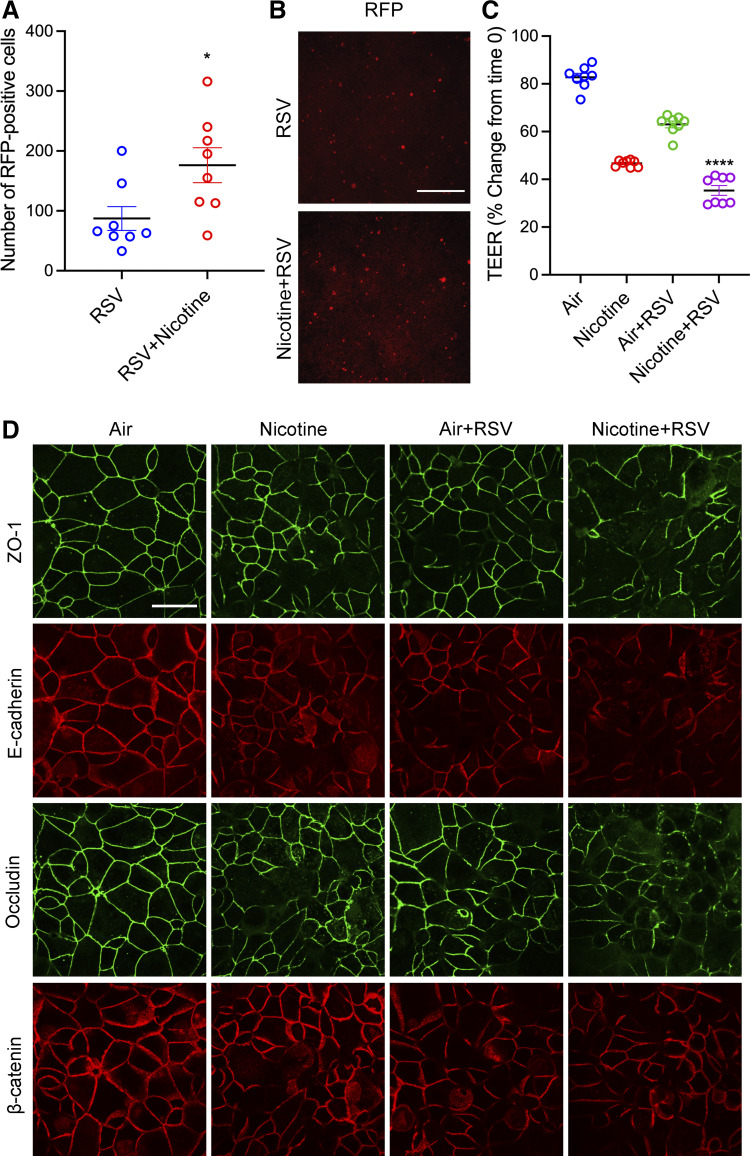
Pre-exposure to aerosolized e-cig nicotine increased RSV-induced airway epithelial barrier disruption. Confluent 16HBE cells were exposed to aerosolized e-cig nicotine and HEPA-filtered air three times for 24 h, followed by RSV infection (rrRSV derived from the RSV A2 strain) at a multiplicity of infection (MOI) of 0.25 for 24 h. *A* and *B*: at 24 h post-RSV infection, the expression of RFP (red fluorescent protein, a representative of viral replication) in infected 16HBE cells was visualized by an inverted fluorescence microscope and quantified by Image J. *C*: TEER (Ω × cm^2^) was measured by the volt-ohm meter and plotted as a percentage change from *time 0*. *D*: 16HBE cells were fixed with methanol and immunolabeled for tight junction proteins (ZO-1 and occludin) and adherens junction proteins (E-cadherin and β-catenin) and imaged by confocal microscopy. Data are presented as means ± SE, *n* = 8, one-way ANOVA followed by Dunnett’s multiple comparisons test. *P < 0.05, ****P < 0.0001. The images are representative of at least three independent experiments. Scale bar, in *B* = 100 μm and *D* = 30 μm. RSV, respiratory syncytial virus; TEER, transepithelial electrical resistance.

RSV infection has been shown to induce airway epithelial barrier dysfunction by mediating TJ and AJ protein disassembly (26, 27, 30, 32). Recent studies have shown that exposure to e-cig chemicals increases the risk of viral infections (11–13, 17, 20, 48). To investigate the role of preexposure to e-cigs on RSV-induced airway barrier dysfunction, we grew 16HBE cell monolayers to confluent and exposed them to HEPA-filtered air or aerosolized e-cig nicotine at 0, 8, and 24 h, followed by RSV (A2 strain, at an MOI of 0.25) or control media for 24 h. Red fluorescent protein, a representative of viral replication, was imaged and counted. Preexposure to aerosolized e-cig nicotine caused a twofold increase in RSV infection (Fig. 7, *A* and *B*). Preexposure to e-cig nicotine significantly exaggerated RSV-induced decrease in TEER (Fig. 7*C*). Furthermore, immunofluorescent staining showed a significant increase in RSV-induced TJ (ZO-1 and occludin) and AJ (E-cadherin and β-catenin) proteins disruption with exposure to e-cig nicotine (Fig. 7*D*).

## DISCUSSION

Tobacco product use is the leading cause of preventable death and disease in the United States (49). E-cigs were introduced to the market in 2007, and since then, there has been an enormous increase in e-cig devices and e-liquid formulations. As such, e-cig use has become a significant public health concern in recent years, increasing users globally, especially teenagers and young adults (50, 51). One critical knowledge gap that needs to be addressed is the health effects of exposure to e-cigs on airway epithelial cells. However, prior studies examining the effects of e-cig exposure in vitro have not extensively investigated the changes in AJC. In the current study, we tested the impact of chemical components of e-cigs on the function and structure of the airway epithelial barrier and viral-induced airway epithelial cell dysfunction. Using our well-characterized in vitro model and a novel custom-built air-tight cell chamber, we analyzed the impact of extract and aerosol form of e-cig components, including PG:VG, nicotine, and flavoring, on the airway epithelial cell barrier. We found that exposure to e-cig chemicals induces a decrease in the integrity of the airway epithelial barrier and disassembly of AJC. We showed preexposure to e-cig nicotine enhanced RSV infection and RSV-induced airway epithelial cell barrier dysfunction. The results of our study provide valuable insights into the respiratory outcomes of e-cig use and highlight the need for further research in this area.

Several approaches have been used to assess the respiratory health effects of e-liquids in vitro, such as direct exposure to e-liquid in the culture medium (52, 53) and exposure to vapor generated from e-liquid by e-cig devices through a variety of commercial or custom-made setups (40, 53–59). Our study used both direct stimulations by e-cig extracts and aerosolized exposures. Exposing the epithelial cells to e-cig liquid by adding those to the cell culture media will help determine the compounds’ stability and solubility. In addition, cell culture exposure via direct stimulation facilitates precise dosage control and rapid analysis of multiple e-liquid formulations on cells of interest, which do not require specialized equipment. In comparison, cell culture exposure via aerosol more precisely simulates physiological e-cig exposure in an in vitro system. Multiple human studies have shown the overall e-cig puff volume is ∼55–110 mL with a duration of 2–3 s and a maximum of 235 puffs per day (34, 35, 37, 60, 61). Based on these parameters, we programmed the ASL 5000 breathing simulator to mimic e-cig puffs and normal breathing patterns of teenagers and connected it to a custom-built novel exposure system for aerosolized e-cig exposures (Fig. 4).

The PG:VG component of e-cigs has generally been perceived as safe by the public; however, recent studies have suggested that PG:VG has adverse effects on the respiratory system, specifically on the viability of airway epithelial cells and the epithelial barrier integrity. For example, it has been reported that exposure to PG:VG in the culture media decreased the viability of A549 cells, and PG:VG aerosol induced cytotoxicity on differentiated primary human bronchial epithelial cells (HBECs) cultured at ALI (40). Consistently, Rowell et al. (53) showed that both PG:VG extract and aerosol caused dose-dependent decreases in cell proliferation and viability in CALU3 cells. Moreover, HBECs exhibited decreased TEER and increased dextran efflux after exposure to PG:VG added to the media (13). In agreement with previous studies, we observed a dose-dependent impact on cell toxicity from PG:VG extract when added to 16HBE cells and found that a higher PG ratio led to increased cytotoxicity (Fig. 1, *E* and *F*). Our data showed that a higher PG ratio in PG:VG mixture induced more disruption to the epithelial monolayer barrier when directly added to culture media and that PG:VG impaired airway epithelial barrier integrity in a concentration-dependent manner (Fig. 1, *A*–*D*). These results joined previous findings in revealing the harmful effects of these commonly used e-cig solvents.

Furthermore, exposure to the nicotine e-liquid or vapor disrupts the airway epithelial integrity of polarized 16HBE cells and differentiated NHBE cells at a nontoxic concentration (0.5–2.5 mM e-liquid or vapor from 36 mg/mL e-liquid) (Figs. 2 and 5). Consistent with our findings, previous studies found that adding e-cig nicotine to the culture media of 16HBE cells led to a rapid and significant decrease in TEER (62). In another study, exposure to e-cig liquid and vapor that contained nicotine increased the permeability of well-differentiated NHBE cells (11). In primary HBECs and Calu-3 cells cultured at ALI, nicotine vapor-infused media has been reported to cause a significant decrease in TEER (63). In addition, we found that exposure to nicotine e-liquid led to dose-dependent cytotoxicity (Fig. 3). Similar to our findings of nicotine-induced cytotoxicity, increased levels of LDH activity following exposure to aerosolized nicotine-rich e-cigs have been observed in the three-dimensional (3-D) culture of human primary nasal epithelial cells (64).

In addition to nicotine, a variety of flavors are added to the e-liquid to increase taste and smell and enhance the consumers’ experience. Currently, more than 14,000 flavored e-liquids are available in the market, classified into 11 main categories (2). The e-cig flavors are mainly made in PG:VG and most likely add to the toxicity of e-cigs (65–67). The addition of flavoring chemicals, including acetoin, maltol, and cinnamaldehyde, directly to the culture media of 16HBE cells has been shown to cause a reduction of TEER (62). Previous studies reported that exposure to nicotine-containing Crème Br«lée and Cool Cucumber flavor aerosols decreased TEER in 16HBE cells (12). In our study, we used two highly marketed flavors from two different categories among adolescents at the time of our study (cinnamon for the spice category and menthol for the mint category). We found that 16HBE cells exhibited decreased TEER and disrupted AJC structure after exposure to aerosols of both flavored e-liquids individually. In addition, coexposure to aerosolized cinnamon flavoring with nicotine increased nicotine-induced barrier disruption (Fig. 6). Given the abundant evidence that e-cig induced cytotoxicity, one mechanism by which inhalation of e-cig chemicals could cause epithelial injury and barrier dysfunction is through cell death and necrosis. However, we observed significant barrier dysfunction and AJC disruption at nontoxic exposure conditions, indicating mechanisms other than cell toxicity. One potential contributor is cellular oxidative stress caused by reactive oxygen species (ROS) generated during the aerosolization of e-liquids (68–70). Indeed, it has been reported that inhibiting ROS could protect epithelial barrier disruption from TiO_2_-NP stimuli (26). It is of particular importance to interrogate the underlying mechanisms in future studies.

Previous epidemiological studies reported that exposure to e-cigs increases the risk of subsequent viral infections (17, 19). In addition, studies using primary tracheobronchial epithelial cells from young, healthy smokers showed increased sensitivity to human rhinovirus infection (20). Our results demonstrated that exposure to e-cig nicotine aerosol exacerbates RSV-induced barrier disruption and promotes viral infection (Fig. 7). An increasing body of literature suggests that viral dissemination can be facilitated by barrier disruption (71–74). However, further studies are needed to fully understand the underlying molecular mechanisms.

There are a few limitations to consider in this study. The current study focuses on nicotine, PG:VG, and flavors of the e-cig components. There has also been a rise in the vaping of non-nicotine substances such as tetrahydrocannabinol (THC), cannabidiol (CBD), and vitamin E acetate (75). A study detected traces of vitamin E acetate in bronchoalveolar lavage (BAL) samples from 48 out of 51 patients from 16 states as a potential chemical involved in the pathology of EVALI (3, 76). In addition, we have not compared higher VG levels in e-liquid. Studies have shown e-cig liquid containing more PG increased the delivery of nicotine and flavor and provided a better throat hit, whereas VG provided a bigger cloud of smoke (77, 78). One study has also stated that higher PG levels produce an unpleasing taste (77). Thus, it is important to perform further studies to compare the impact of higher levels of VG on airway barrier structure and function. Our study does not explore the importance of pharmacological agents in reversing e-cig-induced barrier damage through the reorganization of TJ and AJ proteins (79–82). However, it would be beneficial to design experiments to further the knowledge of restoring the epithelial barrier by using specific antagonists.

Furthermore, in the current study, we have not compared the effect of e-cig exposure to conventional CS or dual exposures. E-cig vaping is advertised as less harmful than conventional CS. Previous studies have shown that in animal models, the impact of e-cig vapor on airway hyperresponsiveness was comparable to CS (83). However, other studies have indicated conflicting results on the effect of e-cigs compared with conventional CS on cell toxicity, morphology, and proliferation (56, 84). Therefore, it is crucial to perform studies to compare the impact of e-cigs with conventional CS, specifically on airway barrier structure and function. Furthermore, in vivo animal studies are essential to validate our in vitro findings in a whole organism.

Taken together, this study highlights the importance of considering e-cig use as a potential risk factor for respiratory infections and the need for more research on the long-term effects of e-cig use on lung health and disease. Moreover, this study provides additional evidence for the rising concerns about using flavored e-cig products. Furthermore, understanding the role of e-cigs on RSV-induced barrier dysfunction will prepare the ground for future studies to determine the effect of other viruses, such as SARS-CoV-2.

## DATA AVAILABILITY

Data will be made available upon reasonable request.

## GRANTS

This work was supported by the National Institutes of Health (National Heart, Lung, and Blood Institute Grant R01HL148057, to F.R.), the Cleveland Clinic Research Program Committee (RPC)-RPC 7524 and Cleveland Clinic Research Program Committee (RPC)-RPC 4159 awards (to F.R). This work utilized the Leica SP8 confocal microscope that was purchased with funding from National Institutes of Health SIG grant 1S10OD019972-01.

## DISCLOSURES

No conflicts of interest, financial or otherwise, are declared by the authors.

## AUTHOR CONTRIBUTIONS

A.R., N.G., R.L.C., and F.R. conceived and designed research; A.R., N.G. and F.R. performed experiments; A.R., N.G., R.L.C., and F.R. analyzed data; A.R., N.G., R.L.C., and F.R. interpreted results of experiments; A.R., N.G., and F.R. prepared figures; A.R., N.G., and F.R. drafted manuscript; A.R., N.G., R.L.C., and F.R. edited and revised manuscript; A.R., N.G., R.L.C., and F.R. approved final version of manuscript.
